# Supplementary figures and images for: Comparative analysis of codon usage patterns and phylogenetic implications of five mitochondrial genomes of the genus Japanagallia Ishihara, 1955 (Hemiptera, Cicadellidae, Megophthalminae)
Source: PeerJ. 2023 Sep 25;11:e16058. doi: 10.7717/peerj.16058 (PMC10538298; doi:10.7717/peerj.16058)

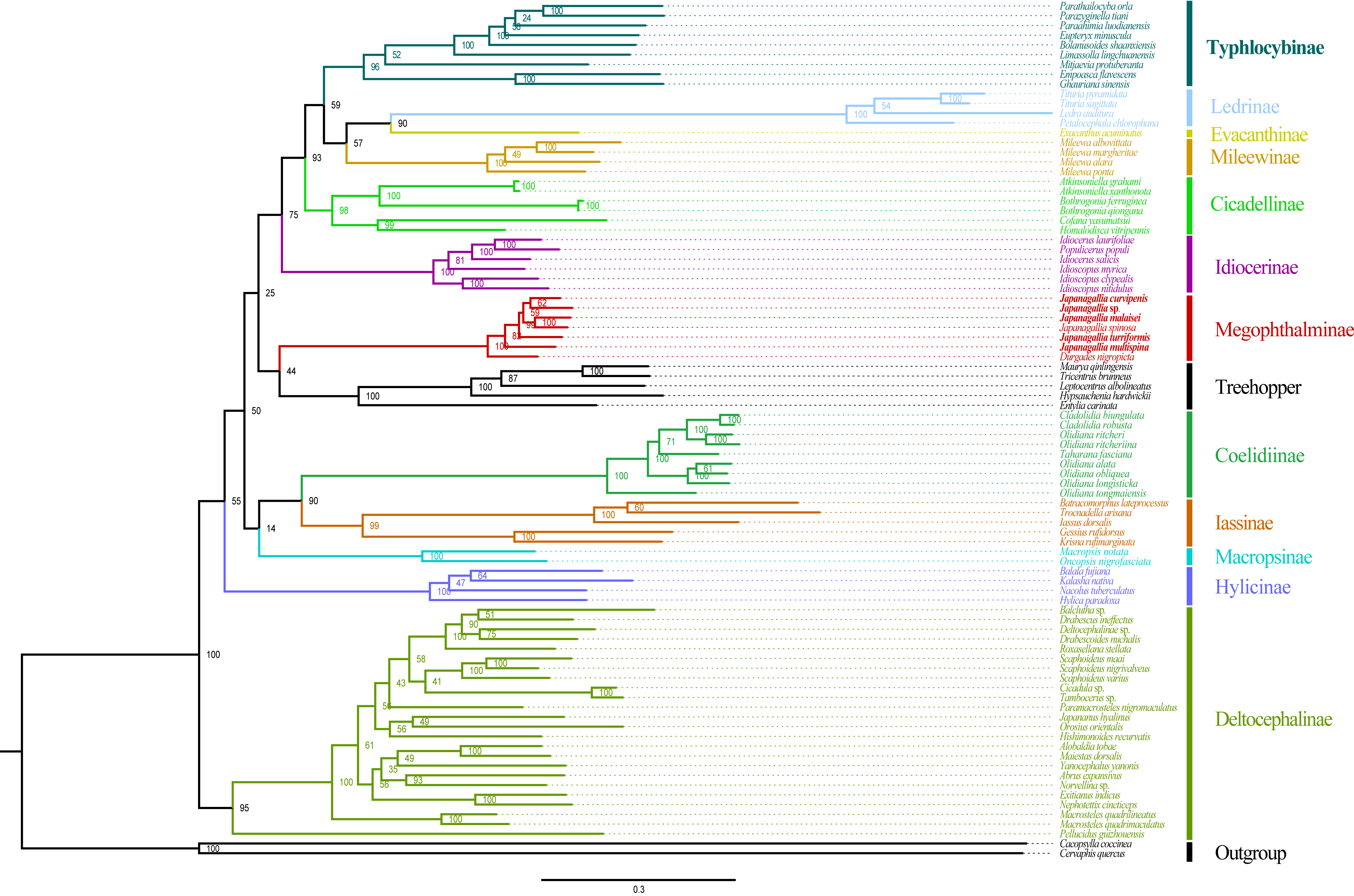

Supplement: Supplemental Information 2 [file peerj-11-16058-s002.png]

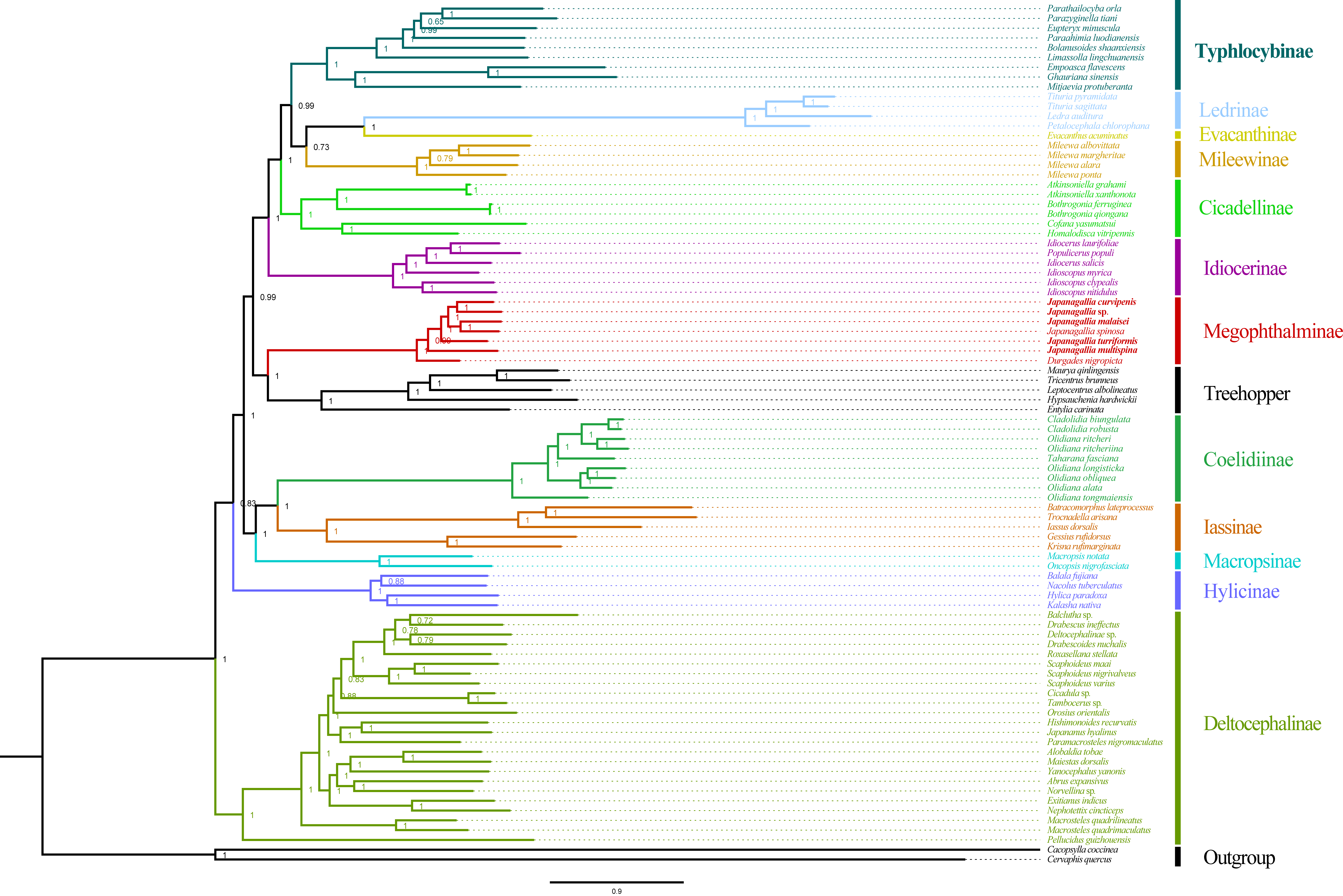

Supplement: Supplemental Information 3 [file peerj-11-16058-s003.png]

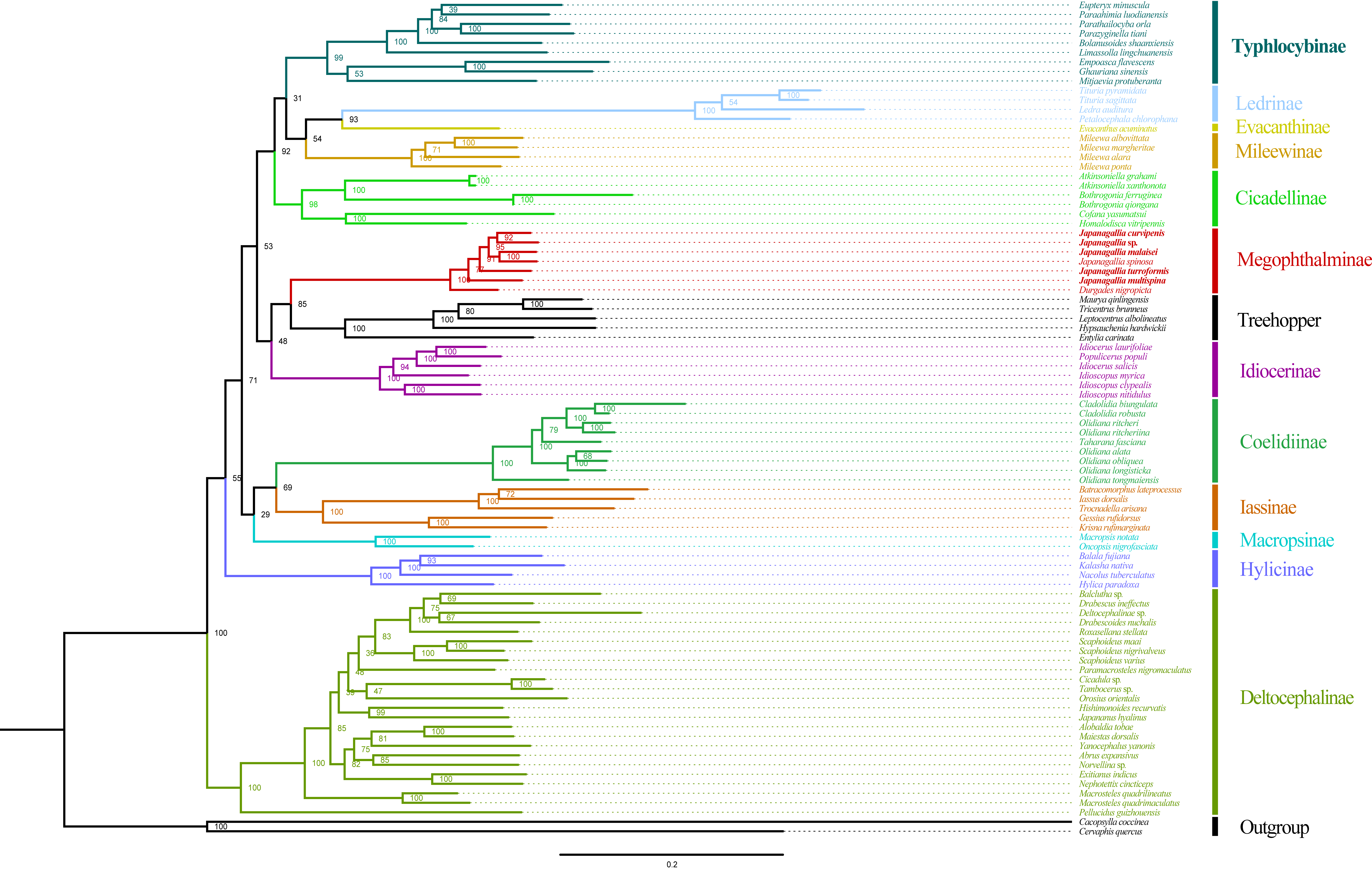

Supplement: Supplemental Information 4 [file peerj-11-16058-s004.png]

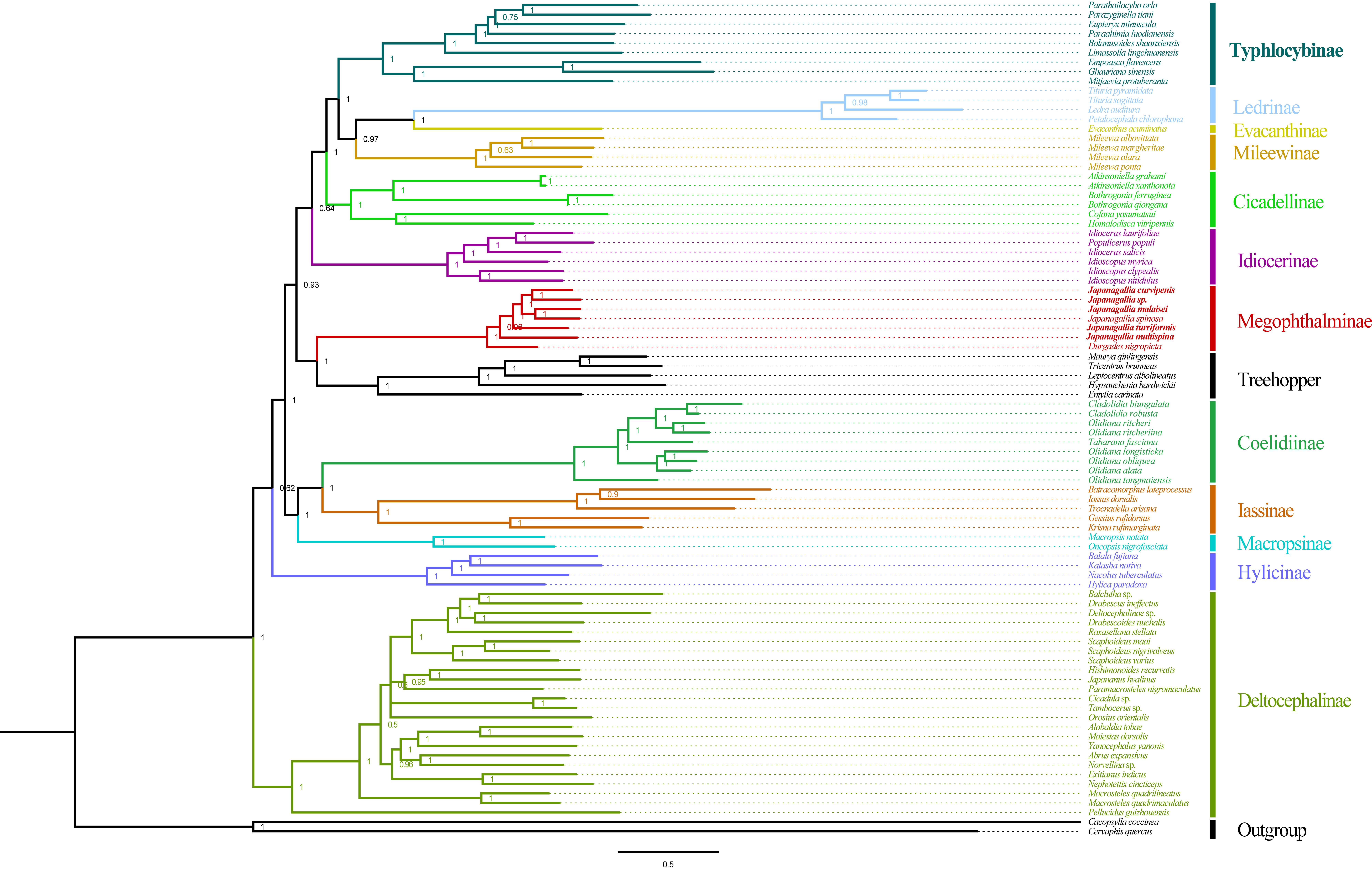

Supplement: Supplemental Information 5 [file peerj-11-16058-s005.png]

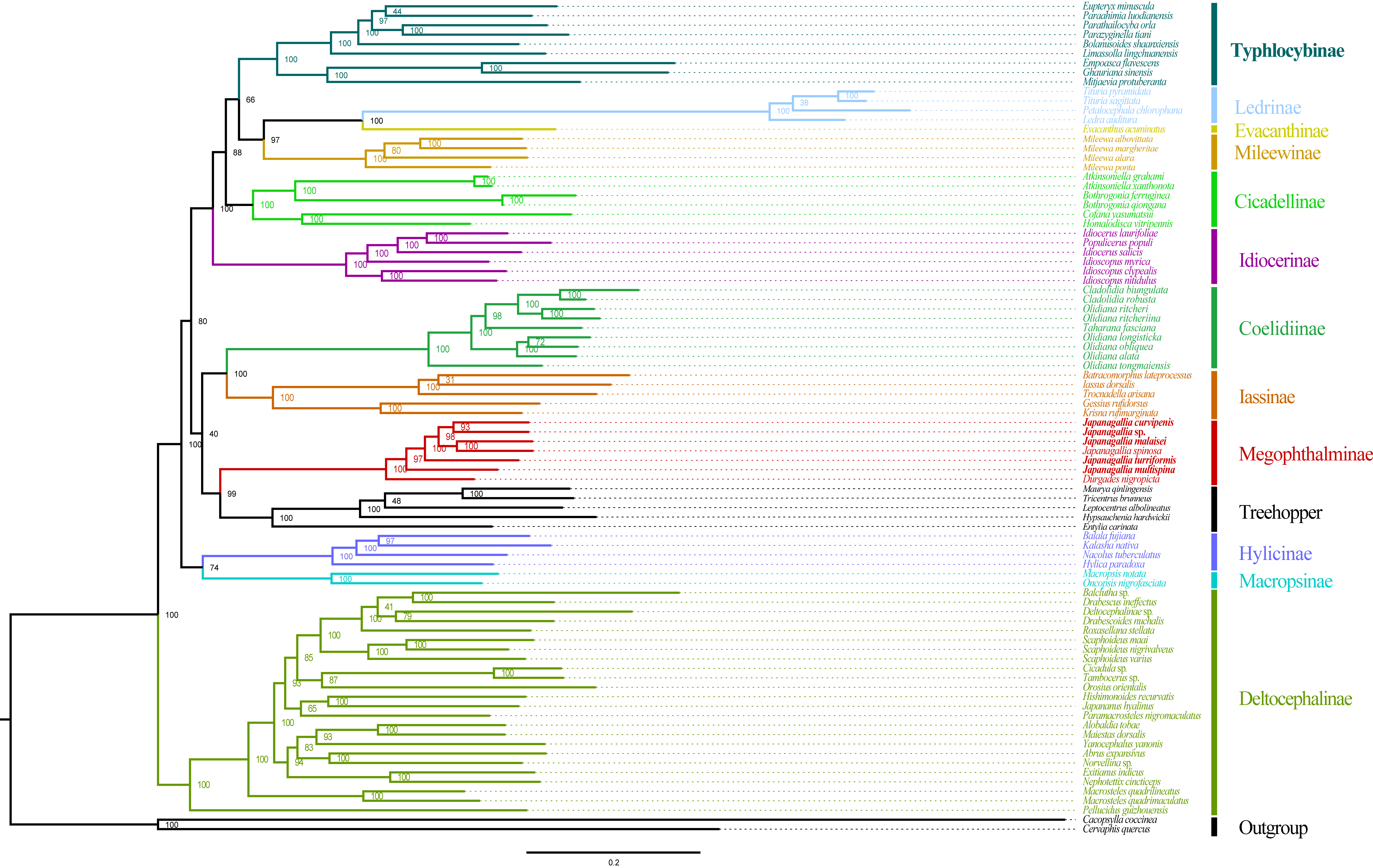

Supplement: Supplemental Information 6 [file peerj-11-16058-s006.png]
