# Supplementary material for: Comparative analysis of codon usage patterns and phylogenetic implications of five mitochondrial genomes of the genus Japanagallia Ishihara, 1955 (Hemiptera, Cicadellidae, Megophthalminae)
Source: PeerJ. 2023 Sep 25;11:e16058. doi: 10.7717/peerj.16058 (PMC10538298; doi:10.7717/peerj.16058)
Supplement: Supplemental Information 7 [file peerj-11-16058-s007.pdf]

Table S1. Mitochondrial genomes used for the phylogenetic analyses in this study

Subfamily	Species	Length (bp)	A+T (%)	GenBank accession no.
Typhlocybinae	*Paraahimia luodianensis*	15,382	79.1%	MN894531
	Parazyginella tiani	17,562	76.4%	MT683891
	Paraahimia luodianensis	16,497	80%	NC_047464
	Eupteryx minusula	16,944	78.8%	MN910279
	Bolanusoides shaanxiensis	15,274	78.9%	MN661136
	Limassolla lingchuanensis	15,716	78.8%	NC_046037
	Mitjaevia protuberanta	15,472	77.4%	NC_047465
	Empoasca flavescens	15,152	78.4%	MK211224
	Ghauriana sinensis	15,491	79.5%	MN699874
Ledrinae	*Tituria pyramidata*	15,331	75.6%	NC_046701
	Tituria sagittata	14,918	76.5%	NC_051528
	Petalocephala chlorophana	14,927	76.6%	MT610899
	Ledra auditura	16,094	76.3%	MK387845
Evacanthinae	*Evacanthus acuminatus*	14,793	78.8%	MK948205
Mileewinae	*Mileewa margheritae*	15,375	79%	MT483998
	Mileewa ponta	15,999	79.9%	MT497465
	Mileewa albovittata	15,079	79.6%	MK138358
	Mileewa alara	16,020	77.9%	MW533151
Cicadellinae	*Bothrogonia ferruginea*	15,262	76.4%	KU167550
	Homalodisca vitripennis	15,304	78.4%	NC_006899
	Atkinsoniella grahami	15,621	78.5%	MW533712
	Atkinsoniella xanthonota	15,895	78.5%	MW533713
	Bothrogonia qiongana	15,788	76.9%	NC_049894
	Cofana yasumatsui	16,011	77.6%	NC_044706
Megophthalminae	*Durgades nigropicta*	15,974	78.8%	NC_035684
	Japanagallia spinosa	15,655	76.6%	NC_035685
	Japanagallia curvipenis	15,356	76.3%	This Study
	Japanagallia malaisei	15,575	77.1%	This Study
	Japanagallia multispina	15,533	77.2%	This Study
	Japanagallia turriformis	15,717	76.1%	This Study
	Japanagallia sp.	15,396	77.7%	This Study
Treehopper	*Maurya qinlingensis*	16,011	78.1%	NC_044706
	Entylia carinata	15,662	78.1%	NC_033539
	Tricentrus brunneus	16,467	78.6%	NC_044708
	Leptocentrus albolineatus	15,508	78.1%	NC_044707
	Hypsauchenia hardwickii	15,618	78.8%	NC_044705
Idiocerinae	*Populicerus populi*	16,494	77.2%	MH492318
	Liocratus salicis	16,436	77.2%	MG813490
	Idioscopus myrica	15,423	77.8%	MH492317
	Idioscopus clypealis	15,393	78.3%	MF784430
	Idiocerus laurifoliae	16,811	79.5%	MH433622
	Idioscopus nitidulus	15,287	78.7%	NC_029203

Coelidiinae	*Olidiana ritcheriina*	15,166	78%	MK738125
	Taharana fasciana	15,161	77.9%	KY886913
	Olidiana alata	15,205	78%	MN780581
	Olidiana longsticka	15,993	79.7%	MN780582
	Olidiana olbliquea	15,312	79.3%	MN780583
	Olidiana ritcheri	15,372	78.2%	MN780584
	Cladolidia biungulata	15,247	78.2%	MW406474
	Cladolidia robusta	15,376	78.4%	MW406475
	Olidiana tongmaiensis	15,363	78.1%	NC_057966
Iassinae	*Batracomorphus lateprocessus*	15,356	80.5%	MG813489
	Trocnadella arisana	15,131	80.7%	NC_036480
	Iassus dorsalis	15,176	80.1%	MN577634
	Krisna rufimarginata	14,724	81.1%	NC_046068
	Gessius rufidorsus	14,634	80.7%	MN577633
Macropsinae	*Macropsis notata*	16,323	76.8%	NC_042723
	Oncopsis nigrofasciata	15,927	79%	MG813492
Hylicinae	*Balala fujiana*	16,221	77.4%	NC_056921
	Kalasha nativa	15,716	74.4%	NC_056922
	Nacolus tuberculatus	15,737	77.1%	NC_056923
	Hylica paradoxa	14,762	75.8%	NC_056920
Deltocephaline	*Drabescus ineffectus*	15,744	77.1%	NC_050258
	Roxasellana stellata	15,361	76%	NC_050257
	Scaphoideus varius	15,207	75.9%	KY817245
	Paramacrosteles nigromaculatus	15,011	76.3%	NC_045270
	Abrus expansivus	15,904	74.7%	NC_045238
	Yanocephalus yanonis	15,623	74.6%	NC_036131
	Maieatas dorsalis	15,352	78.7%	NC_036296
	Macrosteles quadrilineatus	16,626	78%	NC_034781
	Alobaldia tobae	16,026	77.8%	KY039116
	Drabescoides nuchalis	15,309	75.7%	NC_028154
	Exitianus indicus	16,089	75.1%	KY039128
	Japananus hyalinus	15,364	76.6%	NC_036298
	Orosius orientalis	15,513	72%	KY039146
	Scaphoideus nigrivalveus	15,235	76.5%	KY817244
	Pellucidus guizhouensis	16,555	78%	MF784429
	Hishimonoides recurvatis	14,614	76.7%	KY364883
	Scaphoideus maai	15,188	77.2%	KY817243
	Macrosteles quadrimaculatus	15,734	77.7%	NC_039560
	Nephotettix cincticeps	14,805	77.7%	NC_026977
	Deltocephalinae sp.	14,573	74.1%	KX437726
	Cicadula sp.	14,929	74.6%	KX437724
	Norvellina sp.	15,594	74.1%	KY039131
	Tambocerus sp.	15,955	76.4%	KT827824
	Balclutha sp.	14,819	75.8%	KX437738

outgroup	*Cervaphis quercus*	15,272	84.8%	NC_024926
	Cacopsylla coccinea	14,832	72.1%	NC_027087
