# Supplementary material for: Comparative analysis of codon usage patterns and phylogenetic implications of five mitochondrial genomes of the genus Japanagallia Ishihara, 1955 (Hemiptera, Cicadellidae, Megophthalminae)
Source: PeerJ. 2023 Sep 25;11:e16058. doi: 10.7717/peerj.16058 (PMC10538298; doi:10.7717/peerj.16058)
Supplement: Supplemental Information 8 [file peerj-11-16058-s008.pdf]

Table S2. Collection information of specimen in the present study.

**Name**	**Locality**	**Collecter**	**Time**
J. malaisei	Pianma, Lushui, Yunnan (N. 26°10', E. 98°38')	Wang-Jiajia, Zhang-Chao	26, May 2019
J. curvipenis	Yunkai Mountain, Xinyi, Guangdong (N. 22°15', E. 111°38')	Xu-Tielong	25, Apr 2019
J. multispina	Baihua Mountain, Baoshan, Yunnan (N. 25°12', E. 99°18')	Wang-Jiajia, Zhang-Chao	23, May 2019
J. turriformis	Siluo Mountain, Lingshui, Hainan (N. 18°39', E. 109°44')	Wang-Jiajia	18, Aug 2019
Japanagallia. sp.	Pianma, Lushui, Yunnan (N. 26°10', E. 98°38')	Wang-Jiajia, Zhang-Chao	26, May 2019

Note: *J. malaise* indicates *Japanagallia malaise*, *J. curvipenis* indicates *Japanagallia curvipenis*, *J. multispina* indicates *Japanagallia multispina*, *J. turriformis* indicates *Japanagallia turriformis* and *Japanagallia* sp. indicates *Japanagallia* sp.
