# Supplementary material for: Comparative analysis of codon usage patterns and phylogenetic implications of five mitochondrial genomes of the genus Japanagallia Ishihara, 1955 (Hemiptera, Cicadellidae, Megophthalminae)
Source: PeerJ. 2023 Sep 25;11:e16058. doi: 10.7717/peerj.16058 (PMC10538298; doi:10.7717/peerj.16058)
Supplement: Supplemental Information 9 [file peerj-11-16058-s009.pdf]

Table S3. Nucleotide composition analysis in the coding sequences of 13 mitochondrial genes from different mitochondrial genome of five *Japanagallia* species.

ATP6 genes

CDS. NO.	A %	T %	G %	C %	Total bp	A3 %	T3 %	G3 %	C3 %	AT %	GC %	GC1 %	GC2 %	GC3 %	AT3 %	GC12 %
1	34.19	43.51	14.05	8.24	740.00	36.18	41.06	13.41	9.35	72.75	22.30	28.77	34.70	22.76	77.24	31.74
2	35.41	42.84	13.92	7.84	740.00	32.93	47.15	10.98	8.94	78.24	21.76	26.15	33.03	19.92	80.08	29.59
3	35.59	42.63	13.80	7.98	739.00	39.02	40.65	12.60	7.72	78.21	21.79	26.15	32.57	20.33	79.67	29.36
4	34.59	43.92	13.51	7.97	740.00	32.93	45.12	13.41	8.54	78.51	21.49	26.48	34.25	21.95	78.05	30.37
5	33.06	42.91	15.38	8.64	741.00	32.79	43.32	15.79	8.10	75.98	24.02	28.31	31.96	23.89	76.11	30.14
**Mean**	34.57	43.16	14.14	8.14		34.77	43.46	13.24	8.53	76.74	22.27	27.17	33.30	21.77	78.23	30.24
**SD**	0.91	0.48	0.65	0.28		2.48	2.45	1.56	0.58	2.19	0.91	1.13	1.03	1.48	1.48	0.83

ATP8 genes

CDS. NO.	A %	T %	G %	C %	Total bp	A3 %	T3 %	G3 %	C3 %	AT %	GC %	GC1 %	GC2 %	GC3 %	AT3 %	GC12 %
1	46.41	35.95	5.88	11.76	153.00	49.02	39.22	3.92	7.84	82.35	17.65	15.69	25.49	11.76	88.24	20.59
2	49.02	33.99	4.58	12.42	153.00	56.86	33.33	0.00	9.80	83.01	16.99	15.69	25.49	9.80	90.20	20.59
3	49.02	33.33	3.92	13.73	153.00	58.82	33.33	0.00	7.84	82.35	17.65	11.76	33.33	7.84	92.16	22.55
4	50.33	35.29	3.92	10.46	153.00	58.82	33.33	0.00	7.84	85.62	14.38	9.80	25.49	7.84	92.16	17.65
5	46.41	35.29	5.23	13.07	153.00	52.94	33.33	0.00	13.73	81.70	18.30	15.69	25.49	13.73	86.27	20.59
Mean	48.24	34.77	4.71	12.29		55.29	34.51	0.78	9.41	83.01	16.99	13.73	27.06	10.20	89.80	20.39
SD	1.57	0.96	0.76	1.12		3.80	2.35	1.57	2.29	1.37	1.37	2.48	3.14	2.29	2.29	1.57

COX1 genes

CDS. NO.	A %	T %	G %	C %	Total bp	A3 %	T3 %	G3 %	C3 %	AT %	GC %	GC1 %	GC2 %	GC3 %	AT3 %	GC12 %
1	31.84	36.20	14.58	17.38	1536.00	43.75	38.28	5.27	12.70	68.03	31.97	39.06	38.87	17.97	82.03	38.96
2	33.66	35.49	13.70	17.16	1533.00	48.14	35.62	2.94	13.31	69.15	30.85	37.57	38.75	16.24	83.76	38.16
3	33.01	36.53	14.29	16.18	1533.00	45.79	37.57	5.28	11.35	69.54	30.46	36.20	38.55	16.63	83.37	37.38
4	33.01	36.91	14.00	16.08	1536.00	44.92	40.82	4.69	9.57	69.92	30.08	37.30	38.67	14.26	85.74	37.99
5	31.97	35.81	14.84	17.38	1536.00	42.97	36.72	6.84	13.48	67.77	32.23	37.89	38.48	20.31	79.69	38.18
**Mean**	32.69	36.19	14.28	16.84		45.11	37.80	5.00	12.08	68.88	31.12	37.61	38.66	17.08	82.92	38.13
**SD**	0.69	0.51	0.41	0.58		1.80	1.75	1.26	1.46	0.84	0.84	0.92	0.14	2.01	2.01	0.51

COX2 genes

CDS. NO.	A %	T %	G %	C %	Total bp	A3 %	T3 %	G3 %	C3 %	AT %	GC %	GC1 %	GC2 %	GC3 %	AT3 %	GC12 %
1	36.82	32.84	12.37	17.97	679.00	47.35	31.42	6.64	6.64	69.66	30.34	36.12	33.63	13.27	78.76	34.88
2	38.88	34.32	10.46	16.35	679.00	50.44	36.28	1.77	1.77	73.20	26.80	33.48	33.63	3.54	86.73	33.55
3	40.21	34.02	9.87	15.91	679.00	53.10	34.07	0.88	0.88	74.23	25.77	32.16	32.30	1.77	87.17	32.23
4	39.32	33.43	9.72	17.53	679.00	54.87	30.97	1.33	1.33	72.75	27.25	35.68	31.86	2.65	85.84	33.77
5	38.00	34.76	10.75	16.49	679.00	50.00	36.28	2.65	2.65	72.75	27.25	34.80	33.19	5.31	86.28	33.99
**Mean**	38.65	33.87	10.63	16.85		51.15	33.81	2.65	2.65	72.52	27.48	34.45	32.92	5.31	84.96	33.68
**SD**	1.16	0.67	0.95	0.77		2.60	2.28	2.08	2.08	1.53	1.53	1.46	0.72	4.15	3.13	0.86

COX3 genes

CDS. NO.	A %	T %	G %	C %	Total bp	A3 %	T3 %	G3 %	C3 %	AT %	GC %	GC1 %	GC2 %	GC3 %	AT3 %	GC12 %
1	35.38	35.38	12.69	16.54	780.00	46.92	33.46	6.15	13.46	70.77	29.23	32.31	35.77	19.62	80.38	34.04
2	37.56	35.26	10.90	16.28	780.00	51.54	35.00	1.54	11.92	72.82	27.18	32.31	35.77	13.46	86.54	34.04
3	36.28	36.54	12.18	15.00	780.00	48.46	36.15	5.38	10.00	72.82	27.18	30.77	35.38	15.38	84.62	33.08
4	36.79	35.13	12.05	16.03	780.00	50.77	31.15	4.62	13.46	71.92	28.08	30.38	35.77	18.08	81.92	33.08
5	37.31	34.10	11.28	17.31	780.00	51.92	28.08	2.69	17.31	71.41	28.59	30.38	35.38	20.00	80.00	32.88
**Mean**	36.67	35.28	11.82	16.23		49.92	32.77	4.08	13.23	71.95	28.05	31.23	35.62	17.31	82.69	33.42
**SD**	0.78	0.78	0.65	0.75		1.92	2.88	1.71	2.40	0.80	0.80	0.89	0.19	2.52	2.52	0.51

CYTB genes

CDS. NO.	A %	T %	G %	C %	Total bp	A3 %	T3 %	G3 %	C3 %	AT %	GC %	GC1 %	GC2 %	GC3 %	AT3 %	GC12 %
1	35.88	36.68	11.61	15.83	1137.00	48.55	38.26	3.17	10.03	72.56	27.44	34.04	35.09	13.19	86.81	34.56
2	36.07	35.15	11.29	17.49	1098.00	49.18	33.61	1.91	15.30	71.22	28.78	33.33	35.79	17.21	82.79	34.56
3	38.08	32.28	11.43	18.21	1137.00	53.30	26.91	3.69	16.09	70.36	29.64	34.56	34.56	19.79	80.21	34.56
4	37.64	35.80	10.99	15.57	1137.00	51.45	34.56	2.64	11.35	73.44	26.56	30.87	34.83	13.98	86.02	32.85
5	36.68	33.07	11.79	18.47	1137.00	50.40	28.23	4.22	17.15	69.74	30.26	35.09	34.30	21.37	78.63	34.70
**Mean**	36.88	34.59	11.42	17.11		50.58	32.31	3.13	13.97	71.46	28.54	33.58	34.92	17.11	82.89	34.25
**SD**	0.86	1.66	0.27	1.20		1.69	4.19	0.80	2.79	1.37	1.37	1.47	0.51	3.18	3.18	0.70

ND1 genes

CDS. NO.	A %	T %	G %	C %	Total bp	A3 %	T3 %	G3 %	C3 %	AT %	GC %	GC1 %	GC2 %	GC3 %	AT3 %	GC12 %
1	22.08	52.23	16.14	9.55	942.00	23.25	59.87	13.06	3.82	74.31	25.69	27.39	32.80	16.88	83.12	30.10
2	23.07	52.80	15.77	8.36	945.00	26.03	60.00	10.79	3.17	75.87	24.13	26.03	32.38	13.97	86.03	29.21
3	22.61	53.50	15.29	8.60	942.00	24.20	62.10	11.46	2.23	76.11	23.89	25.48	32.48	13.69	86.31	28.98
4	23.15	54.13	14.90	7.82	933.00	24.44	63.02	10.93	1.61	77.28	22.72	23.15	32.48	12.54	87.46	27.81
5	21.97	52.55	16.77	8.70	942.00	22.93	58.92	13.69	4.46	74.52	25.48	26.11	32.17	18.15	81.85	29.14
**Mean**	22.58	53.04	15.77	8.61		24.17	60.78	11.99	3.06	75.62	24.38	25.63	32.46	15.05	84.95	29.05
**SD**	0.49	0.69	0.65	0.56		1.09	1.53	1.17	1.03	1.09	1.09	1.39	0.21	2.11	2.11	0.73

ND2 genes

CDS. NO.	A %	T %	G %	C %	Total bp	A3 %	T3 %	G3 %	C3 %	AT %	GC %	GC1 %	GC2 %	GC3 %	AT3 %	GC12 %
1	39.32	37.98	8.88	13.83	969.00	50.46	31.27	6.19	12.07	77.30	22.70	21.98	27.86	18.27	81.73	24.92
2	41.28	38.49	8.57	11.66	969.00	54.18	33.44	4.33	8.05	79.77	20.23	21.36	26.93	12.38	87.62	24.15
3	41.69	38.60	8.46	11.25	969.00	53.87	33.44	5.88	6.81	80.29	19.71	19.20	27.24	12.69	87.31	23.22
4	41.59	39.42	8.05	10.94	969.00	54.80	35.91	3.72	5.57	81.01	18.99	18.89	28.79	9.29	90.71	23.84
5	39.22	38.39	9.70	12.69	969.00	51.08	32.51	6.50	9.91	77.61	22.39	21.98	28.79	16.41	83.59	25.39
**Mean**	40.62	38.58	8.73	12.07		52.88	33.31	5.33	8.48	79.20	20.80	20.68	27.93	13.81	86.19	24.30
**SD**	1.11	0.47	0.55	1.06		1.76	1.52	1.10	2.30	1.48	1.48	1.36	0.77	3.17	3.17	0.77

ND3 genes

CDS. NO.	A %	T %	G %	C %	Total bp	A3 %	T3 %	G3 %	C3 %	AT %	GC %	GC1 %	GC2 %	GC3 %	AT3 %	GC12 %
1	37.57	37.57	9.32	15.54	354.00	46.61	28.81	6.78	17.80	75.14	24.86	24.58	25.42	24.58	75.42	25.00
2	38.94	39.78	8.12	13.17	357.00	51.26	35.29	1.68	11.76	78.71	21.29	24.37	26.05	13.45	86.55	25.21
3	42.94	38.14	7.34	11.58	354.00	58.47	31.36	2.54	7.63	81.07	18.93	21.19	25.42	10.17	89.83	23.31
4	39.27	39.83	7.91	12.99	354.00	50.00	38.14	3.39	8.47	79.10	20.90	24.58	26.27	11.86	88.14	25.42
5	37.01	38.98	8.47	15.54	354.00	48.31	28.81	5.08	17.80	75.99	24.01	26.27	22.88	22.88	77.12	24.58
**Mean**	39.14	38.86	8.23	13.76		50.93	32.49	3.89	12.69	78.00	22.00	24.20	25.21	16.59	83.41	24.70
**SD**	2.07	0.89	0.66	1.55		4.09	3.69	1.83	4.39	2.16	2.16	1.65	1.21	5.95	5.95	0.75

ND4 genes

CDS. NO.	A %	T %	G %	C %	Total bp	A3 %	T3 %	G3 %	C3 %	AT %	GC %	GC1 %	GC2 %	GC3 %	AT3 %	GC12 %
1	23.54	54.14	13.59	8.73	1317.00	23.69	61.05	9.79	5.47	77.68	22.32	23.01	28.70	15.26	84.74	25.85
2	23.32	54.97	13.53	8.18	1308.00	23.39	63.30	9.40	3.90	78.29	21.71	23.39	28.44	13.30	86.70	25.92
3	23.24	55.66	13.30	7.80	1308.00	24.77	63.30	8.72	3.21	78.90	21.10	21.33	30.05	11.93	88.07	25.69
4	24.45	54.52	13.06	7.97	1317.00	27.33	61.96	7.52	3.19	78.97	21.03	22.55	29.84	10.71	89.29	26.20
5	23.16	54.21	14.20	8.43	1317.00	23.46	61.50	11.16	3.87	77.37	22.63	24.15	28.70	15.03	84.97	26.42
**Mean**	23.54	54.70	13.54	8.22		24.53	62.22	9.32	3.93	78.24	21.76	22.89	29.15	13.25	86.75	26.02
**SD**	0.47	0.56	0.38	0.33		1.49	0.93	1.20	0.83	0.64	0.64	0.94	0.66	1.76	1.76	0.26

ND4L genes

CDS. NO.	A %	T %	G %	C %	Total bp	A3 %	T3 %	G3 %	C3 %	AT %	GC %	GC1 %	GC2 %	GC3 %	AT3 %	GC12 %
1	23.19	55.07	16.30	5.43	276.00	25.00	58.70	15.22	1.09	78.26	21.74	27.17	21.74	16.30	83.70	24.46
2	26.09	55.43	11.96	6.52	276.00	32.61	60.87	4.35	2.17	81.52	18.48	27.17	21.74	6.52	93.48	24.46
3	23.61	56.25	13.89	6.25	288.00	26.04	61.46	11.46	1.04	79.86	20.14	26.04	21.88	12.50	87.50	23.96
4	25.72	55.07	13.41	5.80	276.00	29.35	59.78	9.78	1.09	80.80	19.20	25.00	21.74	10.87	89.13	23.37
5	22.46	54.71	14.86	7.97	276.00	20.65	60.87	13.04	5.43	77.17	22.83	28.26	21.74	18.48	81.52	25.00
**Mean**	24.21	55.32	14.08	6.39		26.72	60.34	10.78	2.16	79.52	20.48	26.73	21.77	12.93	87.07	24.25
**SD**	1.43	0.52	1.45	0.87		4.05	0.98	3.68	1.69	1.60	1.60	1.11	0.05	4.19	4.19	0.55

ND5 genes

CDS. NO.	A %	T %	G %	C %	Total bp	A3 %	T3 %	G3 %	C3 %	AT %	GC %	GC1 %	GC2 %	GC3 %	AT3 %	GC12 %
1	25.21	52.99	13.50	8.30	1674.00	26.34	60.22	9.14	4.30	78.20	21.80	22.04	29.93	13.44	86.56	25.99
2	25.45	52.63	13.80	8.12	1674.00	25.81	60.04	9.86	4.30	78.08	21.92	22.04	29.57	14.16	85.84	25.81
3	24.79	53.41	13.56	8.24	1674.00	25.45	61.11	8.78	4.66	78.20	21.80	21.51	30.47	13.44	86.56	25.99
4	26.11	53.58	12.60	7.71	1674.00	28.49	62.01	6.63	2.87	79.69	20.31	22.58	28.85	9.50	90.50	25.72
5	25.09	53.17	13.26	8.48	1674.00	25.45	62.01	8.42	4.12	78.26	21.74	22.76	29.93	12.54	87.46	26.34
**Mean**	25.33	53.15	13.35	8.17		26.31	61.08	8.57	4.05	78.48	21.52	22.19	29.75	12.62	87.38	25.97
**SD**	0.44	0.33	0.41	0.26		1.14	0.84	1.08	0.62	0.61	0.61	0.44	0.53	1.64	1.64	0.22

ND6 genes

CDS. NO.	A %	T %	G %	C %	Total bp	A3 %	T3 %	G3 %	C3 %	AT %	GC %	GC1 %	GC2 %	GC3 %	AT3 %	GC12 %
1	46.30	35.60	7.41	10.70	486.00	59.88	30.25	2.47	7.41	81.89	18.11	21.60	22.84	9.88	90.12	22.22
2	46.51	32.95	7.36	13.18	516.00	57.56	27.91	4.65	9.88	79.46	20.54	23.26	23.84	14.53	85.47	23.55
3	47.33	34.98	6.79	10.91	486.00	62.96	29.01	1.85	6.17	82.30	17.70	22.22	22.84	8.02	91.98	22.53
4	47.53	33.95	8.02	10.49	486.00	63.58	27.16	4.32	4.94	81.48	18.52	22.84	23.46	9.26	90.74	23.15
5	46.91	32.30	8.64	12.14	486.00	62.35	24.07	3.70	9.88	79.22	20.78	27.16	21.60	13.58	86.42	24.38
**Mean**	46.91	33.94	7.64	11.50		61.22	27.68	3.41	7.68	80.87	19.13	23.42	22.92	11.06	88.94	23.17
**SD**	0.47	1.22	0.63	1.02		2.24	2.08	1.07	1.98	1.28	1.28	1.95	0.76	2.54	2.54	0.76
