# Supplementary material for: Comparative analysis of codon usage patterns and phylogenetic implications of five mitochondrial genomes of the genus Japanagallia Ishihara, 1955 (Hemiptera, Cicadellidae, Megophthalminae)
Source: PeerJ. 2023 Sep 25;11:e16058. doi: 10.7717/peerj.16058 (PMC10538298; doi:10.7717/peerj.16058)
Supplement: Supplemental Information 10 — a mean values of RSCU based on the synonymous codon usage frequencies; N: Total number of preferred codon; *RSCU < 1; Yellow colour represents over-represented codon (RSCU < 1.6). [file peerj-11-16058-s010.pdf]

Table S4. Overall relative synonymous codon usage (RSCU) values in the coding sequences of 13 mitochondrial genes from different mitochondrial genome of five *Japanagallia* species.

ATP6

**Amino Acid**		**Codon**	**N**	**RSCU[a]**	**Amino Acid**		**Codon**	**N**	**RSCU[a]**
Ala	A	GCU*	4.8	2.46			UUC	4.6	0.78
		GCC	0.8	0.41	Leu	L	UUA*	18.4	3.37
		GCA*	2	1.03			UUG	1.2	0.22
		GCG	0.2	0.1			CUU	5	0.91
Arg	R	CGU*	0.6	1.2			CUC	0.4	0.07
		CGC	0	0			CUA*	7.6	1.39
		CGA*	1.4	2.8			CUG	0.2	0.04
		CGG	0	0	Pro	P	CCU*	4.8	2.09
Asn	N	AAU*	9.6	1.26			CCC	0.2	0.09
		AAC	5.6	0.74			CCA*	4.2	1.83
Asp	D	GAU	0.4	0.8			CCG	0	0
		GAC*	0.6	1.2	Ser	S	UCU*	3.4	1.36
Cys	C	UGU	0.6	0.55			UCC	0.8	0.32
		UGC*	1.6	1.45			UCA*	6.8	2.72
Gln	Q	CAA*	2	2			UCG	1	0.4
		CAG	0	0			AGU	0.6	0.24
Clu	E	GAA*	4	2			AGC	0.2	0.08
		GAG	0	0			AGA*	7.2	2.88
Gly	G	GGU	1.6	0.71			AGG	0	0
		GGC	0.6	0.27	Thr	T	ACU*	5.6	1.2
		GGA*	4.8	2.13			ACC	1.8	0.39
		GGG	2	0.89			ACA*	11.2	2.41
His	H	CAU*	1.6	1.07			ACG	0	0
		CAC	1.4	0.93	Tyr	Y	UAU*	3.8	1.23
le	I	AUU*	17.2	1.58			UAC	2.4	0.77
		AUC	4.6	0.42	Val	V	GUU*	2.4	1.17
Lys	K	AAA*	7.8	1.81			GUC	0	0
		AAG	0.8	0.19			GUA*	5.6	2.73
Met	M	AUA*	27.4	1.81			GUG	0.2	0.1
		AUG	2.8	0.19	Trp	W	UGA*	3.6	1.8
Phe	F	UUU*	7.2	1.22			UGG	0.4	0.2

ATP8									
**Amino Acid**		**Codon**	**N**	**RSCU[a]**	**Amino Acid**		**Codon**	**N**	**RSCU[a]**
Ala	A	GCU	0.2	0.8			UUC	0	0
		GCC	0	0	Leu	L	UUA*	3.2	3.56
		GCA*	0.8	3.2			UUG	0.2	0.22
		GCG	0	0			CUU*	1	1.11
Arg	R	CGU	0	0			CUC	0.2	0.22
		CGC	0	0			CUA	0.8	0.89
		CGA	0	0			CUG	0	0
		CGG	0	0	Pro	P	CCU*	1.4	2.55
Asn	N	AAU*	4.2	1.4			CCC	0.2	0.36
		AAC	1.8	0.6			CCA*	0.6	1.09
Asp	D	GAU*	0.2	2			CCG	0	0
		GAC	0	0	Ser	S	UCU	0	0
Cys	C	UGU	0	0			UCC	0.2	0.57
		UGC	0	0			UCA*	1.4	4
Gln	Q	CAA*	1	2			UCG	0	0
		CAG	0	0			AGU	0	0
Clu	E	GAA	0	0			AGC	0.2	0.57
		GAG	0	0			AGA*	1	2.86
Gly	G	GGU	0	0			AGG	0	0
		GGC	0	0	Thr	T	ACU*	2	2.11
		GGA	0	0			ACC	0.2	0.21
		GGG	0	0			ACA*	1.6	1.68
His	H	CAU	0	0			ACG	0	0
		CAC*	0.2	2	Tyr	Y	UAU	0.8	0.73
le	I	AUU*	5	1.85			UAC*	1.4	1.27
		AUC	0.4	0.15	Val	V	GUU	0	0
Lys	K	AAA*	5.8	1.93			GUC	0	0
		AAG	0.2	0.07			GUA*	0.4	4
Met	M	AUA*	6.6	2			GUG	0	0
		AUG	0	0	Trp	W	UGA*	4	2
Phe	F	UUU*	2.8	2			UGG	0	0

COX1

Amino Acid		Codon	N	RSCU[a]	Amino Acid		Codon	N	RSCU[a]
Ala	A	GCU*	11.2	1.93			UUC	11.6	0.59
		GCC	1	0.17	Leu	L	UUA*	29.8	3.52
		GCA*	10.2	1.76			UUG	2.6	0.31
		GCG	0.8	0.14			CUU	7	0.83
Arg	R	CGU	1.4	0.7			CUC	1	0.12
		CGC	0	0			CUA*	9.4	1.11
		CGA*	6.6	3.3			CUG	1	0.12
		CGG	0	0	Pro	P	CCU*	10.2	1.63
Asn	N	AAU*	15	1.46			CCC	2	0.32
		AAC	5.6	0.54			CCA*	12.4	1.98
Asp	D	GAU*	9.8	1.4			CCG	0.4	0.06
		GAC	4.2	0.6	Ser	S	UCU*	13.4	2.11
Cys	C	UGU*	0.2	2			UCC	4.8	0.76
		UGC	0	0			UCA*	19.8	3.12
Gln	Q	CAA*	7.2	1.8			UCG	1.4	0.22
		CAG	0.8	0.2			AGU	0.8	0.13
Clu	E	GAA*	8.2	1.82			AGC	0.8	0.13
		GAG	0.8	0.18			AGA*	8.6	1.35
Gly	G	GGU*	10.8	1.03			AGG	1.2	0.19
		GGC	0.6	0.06	Thr	T	ACU*	15	1.79
		GGA*	23.8	2.27			ACC	4.4	0.52
		GGG	6.8	0.65			ACA*	13.4	1.6
His	H	CAU*	10.8	1.35			ACG	0.8	0.1
		CAC	5.2	0.65	Tyr	Y	UAU*	10.2	1.15
le	I	AUU*	42.4	1.56			UAC	7.6	0.85
		AUC	12	0.44	Val	V	GUU*	7.8	1.08
Lys	K	AAA*	9	1.76			GUC	1	0.14
		AAG	1.2	0.24			GUA*	18.6	2.58
Met	M	AUA*	40.2	1.82			GUG	1.4	0.19
		AUG	4	0.18	Trp	W	UGA*	12.8	1.71
Phe	F	UUU*	27.4	1.41			UGG	2.2	0.29

COX2									
**Amino Acid**		**Codon**	**N**	**RSCU[a]**	**Amino Acid**		**Codon**	**N**	**RSCU[a]**
Ala	A	GCU*	1.4	1.22			UUC	3.8	0.55
		GCC	0.6	0.52	Leu	L	UUA*	14.8	4.11
		GCA*	2.2	1.91			UUG	0.2	0.06
		GCG	0.4	0.35			CUU	1.4	0.39
Arg	R	CGU*	1.8	1.03			CUC	0.2	0.06
		CGC	0.2	0.11			CUA*	4.8	1.33
		CGA*	4.8	2.74			CUG	0.2	0.06
		CGG	0.2	0.11	Pro	P	CCU*	3.6	1.26
Asn	N	AAU*	6.2	1.02			CCC	1	0.35
		AAC	6	0.98			CCA*	6.4	2.25
Asp	D	GAU*	5	1.25			CCG	0.4	0.14
		GAC	3	0.75	Ser	S	UCU*	4.8	1.78
Cys	C	UGU	0.8	0.8			UCC	1.6	0.59
		UGC*	1.2	1.2			UCA*	10.8	4
Gln	Q	CAA*	7.6	1.9			UCG	0.8	0.3
		CAG	0.4	0.1			AGU	0.6	0.22
Clu	E	GAA*	10.2	1.85			AGC	0.2	0.07
		GAG	0.8	0.15			AGA*	2.8	1.04
Gly	G	GGU	1.2	0.6			AGG	0	0
		GGC	0	0	Thr	T	ACU*	4.4	1.26
		GGA*	6	3			ACC	1.2	0.34
		GGG	0.8	0.4			ACA*	8.2	2.34
His	H	CAU*	4.4	1.63			ACG	0.2	0.06
		CAC	1	0.37	Tyr	Y	UAU*	5.2	1.49
le	I	AUU*	22.4	1.6			UAC	1.8	0.51
		AUC	5.6	0.4	Val	V	GUU*	3.2	1.56
Lys	K	AAA*	8.8	1.96			GUC	0.6	0.29
		AAG	0.2	0.04			GUA*	3.8	1.85
Met	M	AUA*	18.6	1.92			GUG	0.6	0.29
		AUG	0.8	0.08	Trp	W	UGA*	5.8	2
Phe	F	UUU*	10	1.45			UGG	0	0

COX3									
**Amino Acid**		**Codon**	**N**	**RSCU[a]**	**Amino Acid**		**Codon**	**N**	**RSCU[a]**
Ala	A	GCU*	1.2	1.09			UUC	9	0.83
		GCC	0.4	0.36	Leu	L	UUA*	12.6	3.32
		GCA*	2.8	2.55			UUG	0.8	0.21
		GCG	0	0			CUU	3.6	0.95
Arg	R	CGU*	0.8	1.07			CUC	0.4	0.11
		CGC	0	0			CUA*	4.6	1.21
		CGA*	2.2	2.93			CUG	0.8	0.21
		CGG	0	0	Pro	P	CCU*	2.8	1.24
Asn	N	AAU*	8.6	1.3			CCC	0.6	0.27
		AAC	4.6	0.7			CCA*	5.6	2.49
Asp	D	GAU*	3	1.43			CCG	0	0
		GAC	1.2	0.57	Ser	S	UCU*	5.2	1.33
Cys	C	UGU	0	0			UCC	1.6	0.41
		UGC	0	0			UCA*	15.8	4.05
Gln	Q	CAA*	4.6	1.84			UCG	1	0.26
		CAG	0.4	0.16			AGU	0.6	0.15
Clu	E	GAA*	6	1.76			AGC	0.4	0.1
		GAG	0.8	0.24			AGA*	6	1.54
Gly	G	GGU*	4.2	1.04			AGG	0.6	0.15
		GGC	0.6	0.15	Thr	T	ACU*	5.2	1.24
		GGA*	9.8	2.42			ACC	1.4	0.33
		GGG	1.6	0.4			ACA*	9.8	2.33
His	H	CAU*	7.2	1.2			ACG	0.4	0.1
		CAC	4.8	0.8	Tyr	Y	UAU*	5.6	1.3
le	I	AUU*	21.4	1.55			UAC	3	0.7
		AUC	6.2	0.45	Val	V	GUU*	3.2	1.14
Lys	K	AAA*	6.4	1.88			GUC	0.2	0.07
		AAG	0.4	0.12			GUA*	7.4	2.64
Met	M	AUA*	24.2	1.82			GUG	0.4	0.14
		AUG	2.4	0.18	Trp	W	UGA*	11	1.83
Phe	F	UUU*	12.6	1.17			UGG	1	0.17

CYTB									
Amino Acid		Codon	N	RSCU[a]	Amino Acid		Codon	N	RSCU[a]
Ala	A	GCU*	5	1.19			UUC	9.6	0.77
		GCC	1.4	0.33	Leu	L	UUA*	27.6	3.55
		GCA*	9.8	2.33			UUG	1.4	0.18
		GCG	0.6	0.14			CUU	4	0.52
Arg	R	CGU	0.8	0.47			CUC	0.8	0.1
		CGC	0.2	0.12			CUA*	12.6	1.62
		CGA*	4.8	2.82			CUG	0.2	0.03
		CGG	1	0.59	Pro	P	CCU*	6.8	1.36
Asn	N	AAU*	12.8	1.14			CCC	1.2	0.24
		AAC	9.6	0.86			CCA*	11.6	2.32
Asp	D	GAU*	6.4	1.33			CCG	0.4	0.08
		GAC	3.2	0.67	Ser	S	UCU*	6.4	1.78
Cys	C	UGU*	1	1			UCC	2.2	0.61
		UGC*	1	1			UCA*	14.2	3.94
Gln	Q	CAA*	4.8	2			UCG	0.4	0.11
		CAG	0	0			AGU	0.2	0.06
Clu	E	GAA*	6	2			AGC	0.2	0.06
		GAG	0	0			AGA*	4.6	1.28
Gly	G	GGU	3.2	0.56			AGG	0.6	0.17
		GGC	0.4	0.07	Thr	T	ACU*	7.8	1.39
		GGA*	16	2.81			ACC	2.2	0.39
		GGG	3.2	0.56			ACA*	11.8	2.11
His	H	CAU*	4.8	1.07			ACG	0.6	0.11
		CAC	4.2	0.93	Tyr	Y	UAU*	10	1.16
le	I	AUU*	33.4	1.58			UAC	7.2	0.84
		AUC	9	0.42	Val	V	GUU*	3.8	1.17
Lys	K	AAA*	14.2	1.89			GUC	0.2	0.06
		AAG	0.8	0.11			GUA*	8.8	2.71
Met	M	AUA*	32	1.93			GUG	0.2	0.06
		AUG	1.2	0.07	Trp	W	UGA*	11.4	1.93
Phe	F	UUU*	15.2	1.23			UGG	0.4	0.07

ND1									
**Amino Acid**		**Codon**	**N**	**RSCU[a]**	**Amino Acid**		**Codon**	**N**	**RSCU[a]**
Ala	A	GCU*	2.6	3.47			UUC	2.2	0.09
		GCC	0.2	0.27	Leu	L	UUA*	31.6	4.03
		GCA	0.2	0.27			UUG*	9.4	1.2
		GCG	0	0			CUU	4.6	0.59
Arg	R	CGU*	5.2	2.97			CUC	0.2	0.03
		CGC	0.2	0.11			CUA	1.2	0.15
		CGA	0.4	0.23			CUG	0	0
		CGG	1.2	0.69	Pro	P	CCU*	6.2	2.7
Asn	N	AAU*	8.2	1.95			CCC	0.8	0.35
		AAC	0.2	0.05			CCA	2.2	0.96
Asp	D	GAU*	5.4	1.86			CCG	0	0
		GAC	0.4	0.14	Ser	S	UCU*	18.8	3.82
Cys	C	UGU*	14	1.92			UCC	0.6	0.12
		UGC	0.6	0.08			UCA*	5.4	1.1
Gln	Q	CAA	2	0.8			UCG	0.2	0.04
		CAG*	3	1.2			AGU*	5.8	1.18
Clu	E	GAA	4	0.8			AGC	0.2	0.04
		GAG*	6	1.2			AGA*	7	1.42
Gly	G	GGU*	8.4	2.21			AGG	1.4	0.28
		GGC	1.2	0.32	Thr	T	ACU*	5.2	3.71
		GGA	2.4	0.63			ACC	0	0
		GGG	3.2	0.84			ACA	0	0
His	H	CAU*	1.4	2			ACG	0.4	0.29
		CAC	0	0	Tyr	Y	UAU*	23.8	1.93
le	I	AUU*	24.6	1.94			UAC	0.8	0.07
		AUC	0.8	0.06	Val	V	GUU*	11.6	2.61
Lys	K	AAA	1.4	0.34			GUC	1.2	0.27
		AAG*	6.8	1.66			GUA	4	0.9
Met	M	AUA*	8	1.57			GUG	1	0.22
		AUG	2.2	0.43	Trp	W	UGA*	5	1.28
Phe	F	UUU*	44.8	1.91			UGG	2.8	0.72

ND2									
Amino Acid		Codon	N	RSCU[a]	Amino Acid		Codon	N	RSCU[a]
Ala	A	GCU*	3	1.71			UUC	4.6	0.5
		GCC	0.6	0.34	Leu	L	UUA*	26.2	3.29
		GCA*	3.4	1.94			UUG	4.4	0.55
		GCG	0	0			CUU	6	0.75
Arg	R	CGU*	1	2			CUC	1.2	0.15
		CGC	0	0			CUA*	9.6	1.21
		CGA*	1	2			CUG	0.4	0.05
		CGG	0	0	Pro	P	CCU*	3	1.94
Asn	N	AAU*	17.6	1.56			CCC	0.2	0.13
		AAC	5	0.44			CCA*	2.4	1.55
Asp	D	GAU*	0.2	2			CCG	0.6	0.39
		GAC	0	0	Ser	S	UCU*	8	1.52
Cys	C	UGU*	0.8	1.33			UCC	1.8	0.34
		UGC	0.4	0.67			UCA*	19.8	3.77
Gln	Q	CAA*	5	1.92			UCG	0.4	0.08
		CAG	0.2	0.08			AGU	3.4	0.65
Clu	E	GAA*	7.6	1.9			AGC	0.8	0.15
		GAG	0.4	0.1			AGA*	7.8	1.49
Gly	G	GGU	2.2	0.94			AGG	0	0
		GGC	0.8	0.34	Thr	T	ACU*	6.6	1.81
		GGA*	3.2	1.36			ACC	0.8	0.22
		GGG*	3.2	1.36			ACA*	7.2	1.97
His	H	CAU	0.4	0.67			ACG	0	0
		CAC*	0.8	1.33	Tyr	Y	UAU*	6	1.22
le	I	AUU*	31.2	1.68			UAC	3.8	0.78
		AUC	6	0.32	Val	V	GUU*	4.4	1.69
Lys	K	AAA*	16	1.84			GUC	0.6	0.23
		AAG	1.4	0.16			GUA*	5.4	2.08
Met	M	AUA*	48.8	1.82			GUG	0	0
		AUG	4.8	0.18	Trp	W	UGA*	6.4	1.64
Phe	F	UUU*	13.8	1.5			UGG	1.4	0.36

ND3									
Amino Acid		Codon	N	RSCU[a]	Amino Acid		Codon	N	RSCU[a]
Ala	A	GCU	0.2	0.33			UUC	4	0.75
		GCC	0.2	0.33	Leu	L	UUA*	9.8	3.46
		GCA*	2	3.33			UUG	0.6	0.21
		GCG	0	0			CUU	2	0.71
Arg	R	CGU*	0.6	2.4			CUC	0.2	0.07
		CGC	0.2	0.8			CUA*	4.4	1.55
		CGA	0.2	0.8			CUG	0	0
		CGG	0	0	Pro	P	CCU	0.8	0.8
Asn	N	AAU*	2.8	1.17			CCC	0	0
		AAC	2	0.83			CCA*	3.2	3.2
Asp	D	GAU*	1.4	1.4			CCG	0	0
		GAC	0.6	0.6	Ser	S	UCU*	2	1.7
Cys	C	UGU*	0.8	1.6			UCC	0	0
		UGC	0.2	0.4			UCA*	3.6	3.06
Gln	Q	CAA*	1.8	1.8			UCG*	1.2	1.02
		CAG	0.2	0.2			AGU	0.6	0.51
Clu	E	GAA*	2.8	1.87			AGC	0	0
		GAG	0.2	0.13			AGA*	1.8	1.53
Gly	G	GGU	0.4	0.53			AGG	0.2	0.17
		GGC	0	0	Thr	T	ACU*	2	1.33
		GGA*	2.4	3.2			ACC	0.8	0.53
		GGG	0.2	0.27			ACA*	3.2	2.13
His	H	CAU*	0.6	1.2			ACG	0	0
		CAC	0.4	0.8	Tyr	Y	UAU*	2.4	1.26
le	I	AUU*	14	1.49			UAC	1.4	0.74
		AUC	4.8	0.51	Val	V	GUU*	1.2	1.33
Lys	K	AAA*	8	2			GUC	0.2	0.22
		AAG	0	0			GUA*	1.8	2
Met	M	AUA*	12.6	1.97			GUG	0.4	0.44
		AUG	0.2	0.03	Trp	W	UGA*	2.6	1.73
Phe	F	UUU*	6.6	1.25			UGG	0.4	0.27

ND4									
**Amino Acid**		**Codon**	**N**	**RSCU[a]**	**Amino Acid**		**Codon**	**N**	**RSCU[a]**
Ala	A	GCU*	7.4	3.44			UUC	3	0.08
		GCC	0.2	0.09	Leu	L	UUA*	44	4.2
		GCA	0.8	0.37			UUG	9.8	0.94
		GCG	0.2	0.09			CUU	6	0.57
Arg	R	CGU*	3	2.4			CUC	0.6	0.06
		CGC	0.4	0.32			CUA	1.8	0.17
		CGA	1.2	0.96			CUG	0.6	0.06
		CGG	0.4	0.32	Pro	P	CCU*	8.2	2.98
Asn	N	AAU*	12.8	1.94			CCC	0.8	0.29
		AAC	0.4	0.06			CCA	1.8	0.65
Asp	D	GAU*	4.8	1.78			CCG	0.2	0.07
		GAC	0.6	0.22	Ser	S	UCU*	20.6	3.39
Cys	C	UGU*	15.8	1.8			UCC	0.4	0.07
		UGC	1.8	0.2			UCA	4.8	0.79
Gln	Q	CAA*	2	1.33			UCG	1.2	0.2
		CAG	1	0.67			AGU*	15.2	2.69
Clu	E	GAA*	3.2	1.07			AGC	1	0.18
		GAG	2.8	0.93			AGA	2.6	0.46
Gly	G	GGU*	15.2	2.69			AGG	3.8	0.67
		GGC	1	0.18	Thr	T	ACU*	5.2	2.54
		GGA	2.6	0.46			ACC	0.4	0.2
		GGG	3.8	0.67			ACA*	2.2	1.07
His	H	CAU*	7.8	1.95			ACG	0.4	0.2
		CAC	0.2	0.05	Tyr	Y	UAU*	33.8	1.85
le	I	AUU*	40	1.86			UAC	2.8	0.15
		AUC	3	0.14	Val	V	GUU*	12.8	2.37
Lys	K	AAA	3.2	0.86			GUC	0.8	0.15
		AAG*	4.2	1.14			GUA*	7	1.3
Met	M	AUA*	19.4	1.4			GUG	1	0.19
		AUG	8.4	0.6	Trp	W	UGA*	3.6	1.2
Phe	F	UUU*	71.4	1.92			UGG	2.4	0.8

			ND4L					
Amino Acid		Codon	N	RSCU[a]	Amino Acid		Codon	N	RSCU[a]
Ala	A	GCU*	1	4			UUC	0.2	0.04
		GCC	0	0	Leu	L	UUA*	11	3.27
		GCA	0	0			UUG*	3.6	1.07
		GCG	0	0			CUU*	4.4	1.31
Arg	R	CGU*	2	4			CUC	0	0
		CGC	0	0			CUA	1.2	0.36
		CGA	0	0			CUG	0	0
		CGG	0	0	Pro	P	CCU	0	0
Asn	N	AAU*	2	2			CCC	0	0
		AAC	0	0			CCA	0	0
Asp	D	GAU*	1.4	1.4			CCG	0	0
		GAC	0.6	0.6	Ser	S	UCU*	2	2.29
Cys	C	UGU*	3.8	1.9			UCC	0	0
		UGC	0.2	0.1			UCA*	2.2	2.51
Gln	Q	CAA	0	0			UCG	0	0
		CAG	0	0			AGU*	2.2	2.51
Clu	E	GAA*	1.2	1.2			AGC	0.2	0.23
		GAG	0.8	0.8			AGA	0	0
Gly	G	GGU*	2.6	2.48			AGG	0.4	0.46
		GGC	0.2	0.19	Thr	T	ACU*	1	4
		GGA	1	0.95			ACC	0	0
		GGG	0.4	0.38			ACA	0	0
His	H	CAU*	1.8	1.8			ACG	0	0
		CAC	0.2	0.2	Tyr	Y	UAU*	7.8	2
le	I	AUU*	9	1.96			UAC	0	0
		AUC	0.2	0.04	Val	V	GUU*	4.6	3.07
Lys	K	AAA	0.2	0.4			GUC	0.2	0.13
		AAG*	0.8	1.6			GUA	1.2	0.8
Met	M	AUA*	5.6	1.27			GUG	0	0
		AUG	3.2	0.73	Trp	W	UGA*	0.8	1.6
Phe	F	UUU*	10.4	1.96			UGG	0.2	0.4

ND5									
Amino Acid		Codon	N	RSCU[a]	Amino Acid		Codon	N	RSCU[a]
Ala	A	GCU*	8.2	2.73			UUC	3.4	0.09
		GCC	0.6	0.2	Leu	L	UUA*	57	4.09
		GCA*	3.2	1.07			UUG	13.8	0.99
		GCG	0	0			CUU	9.2	0.66
Arg	R	CGU*	3	2.4			CUC	0.2	0.01
		CGC	0	0			CUA	3.2	0.23
		CGA*	1.8	1.44			CUG	0.2	0.01
		CGG	0.2	0.16	Pro	P	CCU*	4.6	2.3
Asn	N	AAU*	23.8	1.89			CCC	0.4	0.2
		AAC	1.4	0.11			CCA*	2.8	1.4
Asp	D	GAU*	11	1.77			CCG	0.2	0.1
		GAC	1.4	0.23	Ser	S	UCU*	30.4	3.45
Cys	C	UGU*	14	1.69			UCC	2.4	0.27
		UGC	2.6	0.31			UCA*	8.8	1
Gln	Q	CAA*	3.2	1.03			UCG	1	0.11
		CAG	3	0.97			AGU*	13	1.48
Clu	E	GAA*	4.4	1.57			AGC	0.6	0.07
		GAG	1.2	0.43			AGA*	9.6	1.09
Gly	G	GGU*	19.8	2.68			AGG	4.6	0.52
		GGC	0.4	0.05	Thr	T	ACU*	11.2	3.11
		GGA	3.4	0.46			ACC	1.2	0.33
		GGG	6	0.81			ACA	1.6	0.44
His	H	CAU*	5	1.92			ACG	0.4	0.11
		CAC	0.2	0.08	Tyr	Y	UAU*	50.2	1.82
le	I	AUU*	43.6	1.92			UAC	5	0.18
		AUC	1.8	0.08	Val	V	GUU*	17.4	2.58
Lys	K	AAA*	4.6	1.12			GUC	1	0.15
		AAG	3.6	0.88			GUA*	7.4	1.1
Met	M	AUA*	28	1.51			GUG	1.2	0.18
		AUG	9.2	0.49	Trp	W	UGA*	6.8	1.36
Phe	F	UUU*	76.4	1.91			UGG	3.2	0.64

ND6									
**Amino Acid**		**Codon**	**N**	**RSCU[a]**	**Amino Acid**		**Codon**	**N**	**RSCU[a]**
Ala	A	GCU	0.4	0.57			UUC	1.6	0.48
		GCC	0	0	Leu	L	UUA*	12.4	3.51
		GCA*	2.2	3.14			UUG	1	0.28
		GCG	0.2	0.29			CUU	2.6	0.74
Arg	R	CGU	0	0			CUC	0.4	0.11
		CGC	0.2	0.8			CUA*	4.8	1.36
		CGA*	0.6	2.4			CUG	0	0
		CGG	0.2	0.8	Pro	P	CCU*	1.2	2.18
Asn	N	AAU*	2.4	1.2			CCC	0.4	0.73
		AAC	1.6	0.8			CCA*	0.6	1.09
Asp	D	GAU*	0.8	1.33			CCG	0	0
		GAC	0.4	0.67	Ser	S	UCU*	2.2	1.24
Cys	C	UGU	0	0			UCC	0.6	0.34
		UGC*	0.2	2			UCA*	7	3.94
Gln	Q	CAA*	3.4	1.89			UCG	0.4	0.23
		CAG	0.2	0.11			AGU	0.2	0.11
Clu	E	GAA*	10	1.92			AGC	0.2	0.11
		GAG	0.4	0.08			AGA*	3.6	2.03
Gly	G	GGU	0.4	0.4			AGG	0	0
		GGC	0.2	0.2	Thr	T	ACU*	4	1.31
		GGA*	3.2	3.2			ACC	1.8	0.59
		GGG	0.2	0.2			ACA*	6	1.97
His	H	CAU*	1.6	1.6			ACG	0.4	0.13
		CAC	0.4	0.4	Tyr	Y	UAU*	2.4	1.14
le	I	AUU*	20.8	1.76			UAC	1.8	0.86
		AUC	2.8	0.24	Val	V	GUU*	1.4	1.65
Lys	K	AAA*	14.4	1.92			GUC	0	0
		AAG	0.6	0.08			GUA*	1.8	2.12
Met	M	AUA*	28.4	1.88			GUG	0.2	0.24
		AUG	1.8	0.12	Trp	W	UGA*	1	2
Phe	F	UUU*	5	1.52			UGG	0	0

[a] mean values of RSCU based on the synonymous codon usage frequencies; N: Total number of preferred codon; *RSCU>1; Yellow colour represents over-represented codon (RSCU>1.6).
